# Integration of Next Generation Sequencing and EPR Analysis to Uncover Molecular Mechanism Underlying Shell Color Variation in Scallops

**DOI:** 10.1371/journal.pone.0161876

**Published:** 2016-08-26

**Authors:** Xiujun Sun, Zhihong Liu, Liqing Zhou, Biao Wu, Yinghui Dong, Aiguo Yang

**Affiliations:** 1 Yellow Sea Fisheries Research Institute, Chinese Academy of Fishery Sciences, Qingdao, China; 2 Zhejiang Provincial Top Key Discipline of Biological Engineering, Zhejiang Wanli University, Ningbo, 315100, China; University of Alabama at Birmingham, UNITED STATES

## Abstract

The Yesso scallop *Patinopecten yessoensis* displays polymorphism in shell colors, which is of great interest for the scallop industry. To identify genes involved in the shell coloration, in the present study, we investigate the transcriptome differences by Illumina digital gene expression (DGE) analysis in two extreme color phenotypes, Red and White. Illumina sequencing yields a total of 62,715,364 clean sequence reads, and more than 85% reads are mapped into our previously sequenced transcriptome. There are 25 significantly differentially expressed genes between Red and White scallops. EPR (Electron paramagnetic resonance) analysis has identified EPR spectra of pheomelanin and eumelanin in the red shells, but not in the white shells. Compared to the Red scallops, the White scallops have relatively higher mRNA expression in tyrosinase genes, but lower expression in other melanogensis-associated genes. Meantime, the relatively lower tyrosinase protein and decreased tyrosinase activity in White scallops are suggested to be associated with the lack of melanin in the white shells. Our findings highlight the functional roles of melanogensis-associated genes in the melanization process of scallop shells, and shed new lights on the transcriptional and post-transcriptional mechanisms in the regulation of tyrosinase activity during the process of melanin synthesis. The present results will assist our molecular understanding of melanin synthesis underlying shell color polymorphism in scallops, as well as other bivalves, and also help the color-based breeding in shellfish aquaculture.

## Introduction

**Fig 1:**
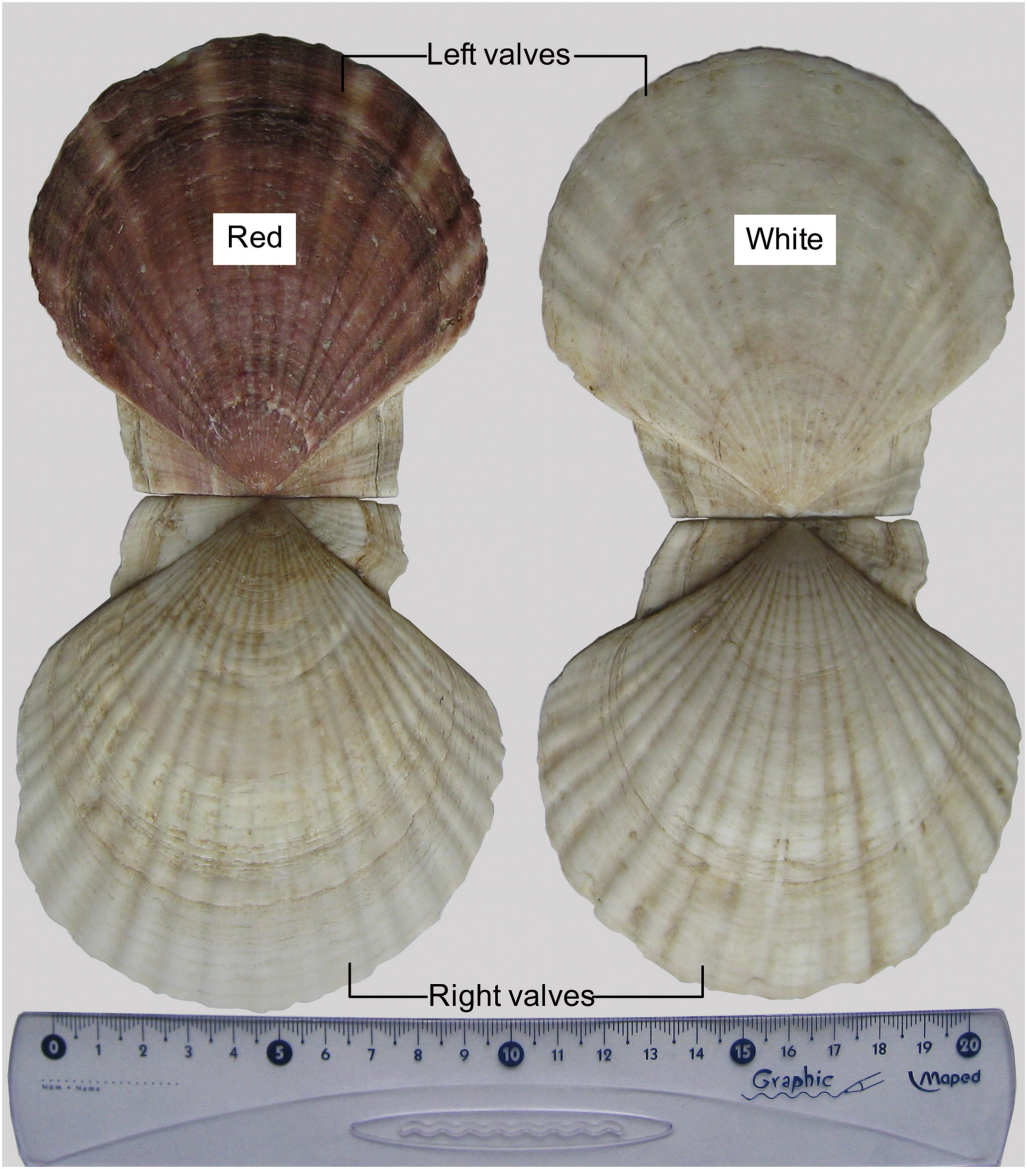
The external view for left and right valves of Yesso scallop *Patinopecten yessoensis*. The predominant color of left valve is reddish-brown or white, while the color of right valve is white.

Yesso scallop (*Patinopecten yessoensis*) is an economically important marine bivalve species in aquaculture and fishery in Asian countries, due to its large and edible adductor muscle [1]. The colors of the left and right valves are obviously distinct, typically having reddish-brown for the left and white for the right. In the cultured populations, we found the rare occurrence of albinism in the left valve, displaying two-side white phenotypes (Fig 1). Due to the higher muscle production of the two-side white individuals, they have been under mass selection since 2004 to produce the new color strain for aquaculture. In land animals, albinism is thought to be caused by the reduction or absence in the synthesis of melanin [2, 3]. However, to date, the cause of the albino phenotype in the scallop *P*. *yessoensis* is still unknown. The availability of albino specimens and normal color specimens is the ideal material to uncover the molecular mechanisms directly linked to shell color variation in this species. In our previous study, the deep sequencing of mantle transcriptome for the scallop has identified some dual functioning proteins involved in both biomineralization and melanogenesis, including proteins related to calcium metabolism, and putative tyrosinase-like protein [4]. Furthermore, we have extracted melanin extract from the pigmented shells in our previous study which allows us to propose the hypothesis that the occurrence of albino specimens is probably due to lack of melanin.

Color polymorphism, where two or more distinct color morphs occur within species, is responsible for recognition, adaption, and camouflage, and has been documented in a variety of taxa in animal kingdom [5–7]. In both land and aquatic animals, color variation has been of great interests to the scientific and breeding communities [8–11]. Molluscs, a large phylum of invertebrate animals, usually show fabulous and diverse colors and patterns in their shells, which have been appreciated for hundreds of years by collectors and scientists [12–14]. Shell color in molluscs is most commonly due to the presence of melanins, indigoids, quinones and flavones [13,15]. Following the pioneering work of Comfort, carotenoids or polyenes were also identified as shell pigments in molluscs by Resonance Raman microspectrometry in more recent studies [16–18]. However, most of the biological pigments in molluscan shells have not been well studied due to difficulties in extraction and highly complex chemistry [15].

As one of those shell pigments, melanin is ubiquitous in nature and produced by a variety of organisms, including bacteria, fungi, plants, and animals [9, 19–21]. In land animals, the colors in feathers, fur and skin are largely determined by melanocytes, which are responsible for melanin production, and melanin is the main contributor to their pigmentation [22]. The quantity, quality, and distribution of melanin contents in animal tissues are usually correlated with the visual phenotypes of their pigmentation [7, 11, 23]. In contrast, the nature of melanin is far from being well understood in molluscs, such as bivalves, although melanin is almost certainly common in their shells [15]. Due to the difficulty in extracting the pigments, melanin in shells has been rarely identified and characterized by chemical methods.

For molluscs, regulatory mechanism for melanin synthesis is poorly understood in bivalves, but it is well known in cephalopods. Cephalopod molluscs have the ink gland to produce melanin, mainly eumelanin, which underscores the complex interplay of melanogenic enzymes and regulatory factors [24, 25]. Studies on melanogenesis in the ink gland of cephalopods have revealed ink production are affected and modulated by neuronal NOS (nitric oxide synthase) and cyclic GMP (cGMP) in certain pathways of central nervous system, which control the activation of tyrosinase and increased melanin synthesis in the gland [25, 26]. Additionally, according to the comprehensive melanin studies in mammals and birds, two main types of melanin (pheomelanin and eumelanin) are both regulated by a common tyrosinase-dependent pathway with the same precursor, tyrosine [7, 9, 22]. All these studies shed lights on the functional role of tyrosinase on melanin production in the ink gland, feather, skin, and hair. However, in bivalve molluscs, mantle tissue is the key organ that secretes proteins responsible for calcification of shells and shell pigments, which seems to have a different melanin-producing system from that in mammals, birds, and cephalopods [15]. For bivalves, the biological pigments probably formed in the secretory cells in the mantle and incorporated into the shells along the growing edge [15, 27–29]. To date, with the rapid development of next generation sequencing (NGS) technology, the transcriptome characterization in the mantle tissue and has enabled us to identify dozens of genes potentially related to shell formation and pigmentation at the transcriptome level [4, 30–33]. The identified proteins and genes will greatly help the understanding of molecular progress for pigment synthesis in mantle and distribution in shell of molluscs. Despite molecular studies related to shell pigmentation are burgeoning, most of these studies do not have the pigments identified biochemically prior to molecular analyses, which may cause some problems in identification of potential genes related to color variation. Therefore, the integration of NGS technology and biochemical studies to explore the potential link between gene expression and color phenotypes is essential to understand shell color polymorphism in molluscs.

To uncover molecular mechanism underlying shell color variation in scallops, in the present study we performed digital gene expression (DGE) analysis to identify differentially expressed genes in mantle of two extreme color phenotypes of *P*. *yessoensis*. Furthermore, EPR (Electron paramagnetic resonance) measurement was performed to identify melanin content in the red and white shells, and explore the relationship between gene expression and melanin content. With the integration of NGS technology, EPR, qPCR, ELISA and enzyme activity, the present findings highlight the functional roles of melanogensis-associated genes in the melanization process of scallop shells, which could help better understand the molecular mechanism underlying shell color variation in scallops, as well as other molluscs.

## Methods

### Ethics Statement

The scallops used in the current study are marine-cultured animals, and all the experiments on scallops were conducted following the institutional and national guidelines. No endangered or protected species is involved in the present study, and no specific permission is required for the location of the culture experiment.

### Animal and tissue collection

We randomly collected two-year-old live individuals of *P*. *yessoensis*, six from the two-side white strain (the fourth selected generation), termed as “White”, and six from normal color specimens, termed as “Red”. To obtain high quality of gene expression data, all of these scallops were cultured at the same condition, in sand-filtered sea water at 14 ± 2°C with the salinity of 32 ± 2 psu for two weeks, in a commercial hatchery in Jiaonan, China. Scallops were fed with *Isochrysis galbana* and two-thirds of the culture water was exchanged for each day. The mantle tissue of left valves dissected from each scallop was stored in RNAlater individually (Ambion). Total RNA was isolated from collected mantle tissue with Trizol Reagent (Invitrogen) following the manufacturer’s instruction. RNA purity and quality of the samples were checked using the NanoPhotometer^™^ spectrophotometer (Implen, CA, USA). RNA integrity was assessed using the RNA Nano 6000 Assay Kit.

### NGS library construction, sequencing and quality control

RNA samples of three individuals from the same color type were pooled in equal amounts of 3 μg RNA to generate the mixed sample used for the construction of sequencing library. Two biological replicates were used for the Illumina sequencing. The sequencing libraries were generated using NEBNext Ultra^™^ RNA Library Prep Kit for Illumina (NEB, USA) following manufacturer’s recommendations. After mRNA being purified, the first strand cDNA was synthesized using random hexamer primer, and second strand cDNA synthesis was subsequently performed using DNA Polymerase I and RNase H. The detailed method for library construction was according to the previous study [4]. In brief, after the ligation of adaptors, the fragments were purified with AMPure XP system (Beckman Coulter, Beverly, USA). The adaptor-ligated cDNA was used for PCR amplification, and PCR products were purified and library quality was assessed. After cluster generation, the library sequencing was carried out in Illumina HiSeq 2000 platfrom using the 100 bp paired-end strategy.

To ensure the data quality used for the downstream analyses, reads containing ambiguous ‘N’ nucleotides and with quality score of less than 5, was removed from further analysis. After the quality filtering, Q20, Q30 and GC-content of the clean data were calculated for estimating the quality.

### Aligning clean reads and differentially expressed genes analysis

The clean data were then mapped to the previously assembled transcriptome due to lack of genome resources in the studied species [4], using RSEM [34]. The FPKM (reads per kilobase per million reads) was applied to measure the gene expression levels, which were estimated by read counts obtained from the mapping results for each gene. Differential gene expression analysis was performed to uncover the gene expression profiles of the mantle samples from White and Red scallops using the DESeq R package [35]. To minimize the false discovery rates, *P* values were adjusted by the Benjamini and Hochberg’s approach, with a significance of *P* < 0.05.

To verify the data accuracy of NGS sequencing, quantitative real-time PCR (qPCR) was used to compare the relative mRNA expression of significantly expressed genes between the mantle of White and Red scallops. Total RNAs were extracted from the mantle of left valves using Trizol Reagent (Invitrogen). The quality and quantity of total RNA were estimated by ultraviolet spectroscopy using a NanoDrop 2000 spectrophotometer (Thermo Scientific, USA). The degradation and contamination of RNA samples was monitored on 1% agarose gels. Three biological replicates were used for qPCR analysis on Applied Biosystems 7500 system. The comparative Ct method (2^-ΔΔCt^ method) was used to calculate the relative gene expression of the samples, which was normalized to β-actin mRNA level [36]. The expression data were subsequently subjected to one-way ANOVA or independent T-test in SPSS 17.0 to determine whether there was any significant difference with *P* < 0.05.

### Analysis of tyrosinase activities and tyrosinase protein level

Melanin biosynthesis can be initiated from L-tyrosine, which is hydroxylated to L-dihydroxyphenylalanine (L-DOPA) and followed by oxidation of L-DOPA to dopaquinone, showing different absorbance spectra [37]. According to this, the enzyme activity of tyrosinase in the left mantle of Red and White scallops was measured the 475 nm absorbance using Animal tissue tyrosinase activity assay Kit (Haling, Shanghai). The measurements were performed at 25°C according to the manufacturer’s instruction. One unit of enzyme activity was defined as the amount of enzyme required to oxidize 1 μmol substrate within one minute under standard assay conditions. Protein concentration was determined by the Bradford’s method using Bradford Protein Assay Kit (Sangon Biotech). Furthermore, enzyme-linked immunosorbent assay (ELISA) was used for quantitative analysis of tyrosinase in the mantle tissues, which was measured by Fish Tyrosinase ELISA Kit (Jianglaibio, Shanghai) following the manufacturer’s instruction. All the absorbance measurements were performed using three biological replicates on a Multiskan GO Spectrophotometer (Thermo Fisher Scientific, Waltham, MA, USA).

### Melanin extraction from shells and quantification analysis of melanin

The red and white shells were carefully cleaned and dried completely in air. The dried shell samples were grinded into shell powder, which were used as the raw material for melanin extraction. The dry weight of 7 g shell powder was selected and treated by 6 M hydrochloric acid (HCl), and melanin was extracted by hot reflux method according to the previous study [38]. The crude products of melanin were collected by filtration on a Buchner funnel, and purified in a Soxhlet extractor with petroleum ether (60–80°C for 4 h). The melanin was washed and dried until constant weight, and then left in air to equilibrate with moisture for 24 h.

The melanin extracts from Red and White shells were determined using Electron paramagnetic resonance (EPR) measurements [39]. To increase the sensitivity of EPR determination, measurements were carried out at 100K in liquid nitrogen in the presence of 50 mM zinc acetate using Bruker EPR A300 spectrometer [40]. The parameters of EPR measurement were set as follows: microwave power 20 mW, microwave frequency 9 GHz, modulation amplitude 2.0 G, scan range 50 G. The zinc acetate suspension of 10 mg eumelanin (melanin from *Sepia officinalis*, Sigma) and 10 mg synthetic pheomelanin were served as the EPR standards. The synthetic pheomelanin was prepared from L-cysteine (1.5 mmol) and L-Dopa (1.0 mmol) according to the previous study [41] with minor modification. Briefly, L-Dopa and L-cysteine were dissolved in 100 ml of 0.05 M sodium phosphate buffer (pH 6.8). The mixture was incubated at 37°C under oxygen current for 4 h in the presence of 20 mg of mushroom tyrosinase (>500 U/mg, Worthington Biochemical Corporation). The mixture was acidified to pH 3 with acetic acid and kept at 4°C for 1 h. The extract was collected by centrifugation, washed with acetic acid and acetone, and then dried in a vacuum freeze dryer for 8 h. The EPR measurement of melanin samples and standards were operated under identical experimental and apparatus conditions. Estimation of melanin content in terms of eumelanin to pheomelanin (a/b) is based on the comparison of major and additional peak height [42].

## Results

### Overall statistics and reads

There are a total of 63,311,680 raw reads generated by Illumina sequencing. The raw reads have been deposited in the NCBI SRA database (accession number: SRP065869). After quality control (remove reads containing adapters, ambiguous nucleotides and low quality reads), all sequencing data yields a total of 6.27 G clean bases. There are a total of 1.00 G, 1.92 G, 1.68 G, and 1.67 G clean bases generated in the sequencing libraries of Red_123, Red_456, White_123, and White_456, respectively (Table 1). The clean reads are further used for mapping with the reference mantle transcriptome of *P*. *yessoensis* [4]. The sequence alignment indicates that more than 85% reads are mapped into the reference transcriptome. In the four sequencing libraries, the similar values of Q20 percentage are detected among them, all around 98%. The same error rates are observed at 0.03% in the four libraries, and the percentage of GC content varies from 41% to 43%.

**Table 1:** Summary statistics for sequencing and data quality of RNA-Seq.

Sample	Raw Reads	Clean Reads	Clean Bases	Error (%)	Q20 (%)	Q30 (%)	GC Content (%)
C0_123	10,061,486	9,952,872	1.00G	0.03	98.1	93.56	42.17
C0_456	19,330,562	19,176,083	1.92G	0.03	98.22	93.88	41.16
T1_123	16,998,373	16,843,330	1.68G	0.03	98.12	93.56	41.43
T1_456	16,921,259	16,743,079	1.67G	0.03	98.16	93.66	41.93

### Quality control of gene expression analysis

Quality control of gene expression analysis is determined by saturation curves and correlation analysis. Saturation curves display the number of genes detected by uniquely mapped reads with different FPKM values as a function of the sequencing depth. For the blue line, it tracks the number of genes for transcripts with FPKM of 60–150, while the green line tracks the performance for transcripts with FPKM of 3–15. It is indicated that the fraction of transcript increases with additional sequencing data for the high expressed transcripts (FPKM > 3), even at low mapping rates (less than 20%). The expression data is significantly linear correlated between the two replicates of White scallops, as well as Red scallops, both having the squared Pearson correlation coefficient (R^2^) > 0.7 (R^2^ = 0.724 for Red_123 and Red_456; R^2^ = 0.792 for White_123 and White_456).

### Identification of differentially expressed genes and functional annotation

**Fig 2:**
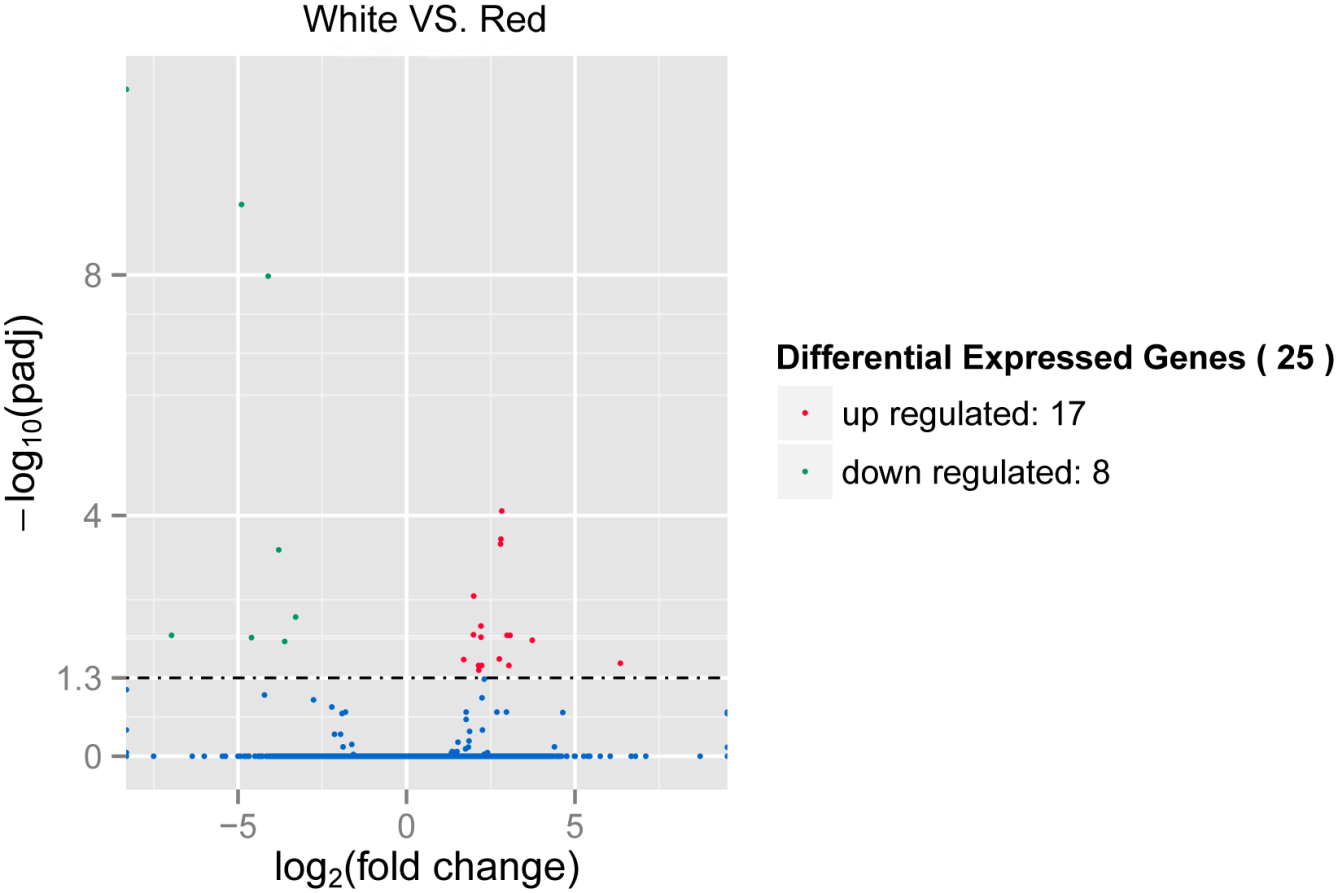
The volcano plots for differentially expressed genes between Red and White. Green points represent transcripts with significantly lower expression level, while red circles represent transcripts with significantly higher expression level (*P* < 0.05).

Identification of different levels of expression for each library sample has pointed out that there are 25 significantly differentially expressed genes between Red and White scallops (Fig 2). Most of the detected genes are not annotated by the NR description, except for comp91333_c0, comp54314_c0, and comp94117_c0, which are identified as Poly [ADP-ribose] polymerase 14, hypothetical protein LOC100494154, and putative tyrosinase-like protein TYR-3, respectively. The unigene of comp94117_c0 is also annotated by PFAM domain description, which has the common central domain of tyrosinase. Additionally, the functional domain of four other unigenes are also detected by PFAM, including SRI (Set2 Rpb1 interacting) domain, Conotoxin, nucleic acid binding, and DNA polymerase (viral) C-terminal domain. Among the 25 screened genes, 17 genes (Red dots) are observed to have significantly higher expression level in White scallops than Red ones, while 8 genes (Green dots) exhibit significantly higher expression level in Red scallops than White ones (Fig 2). For comp94117_c0, the expression of TYR-3 gene in White is almost 4-fold higher than that in Red.

### Gene expression analysis by qPCR

**Fig 3:**
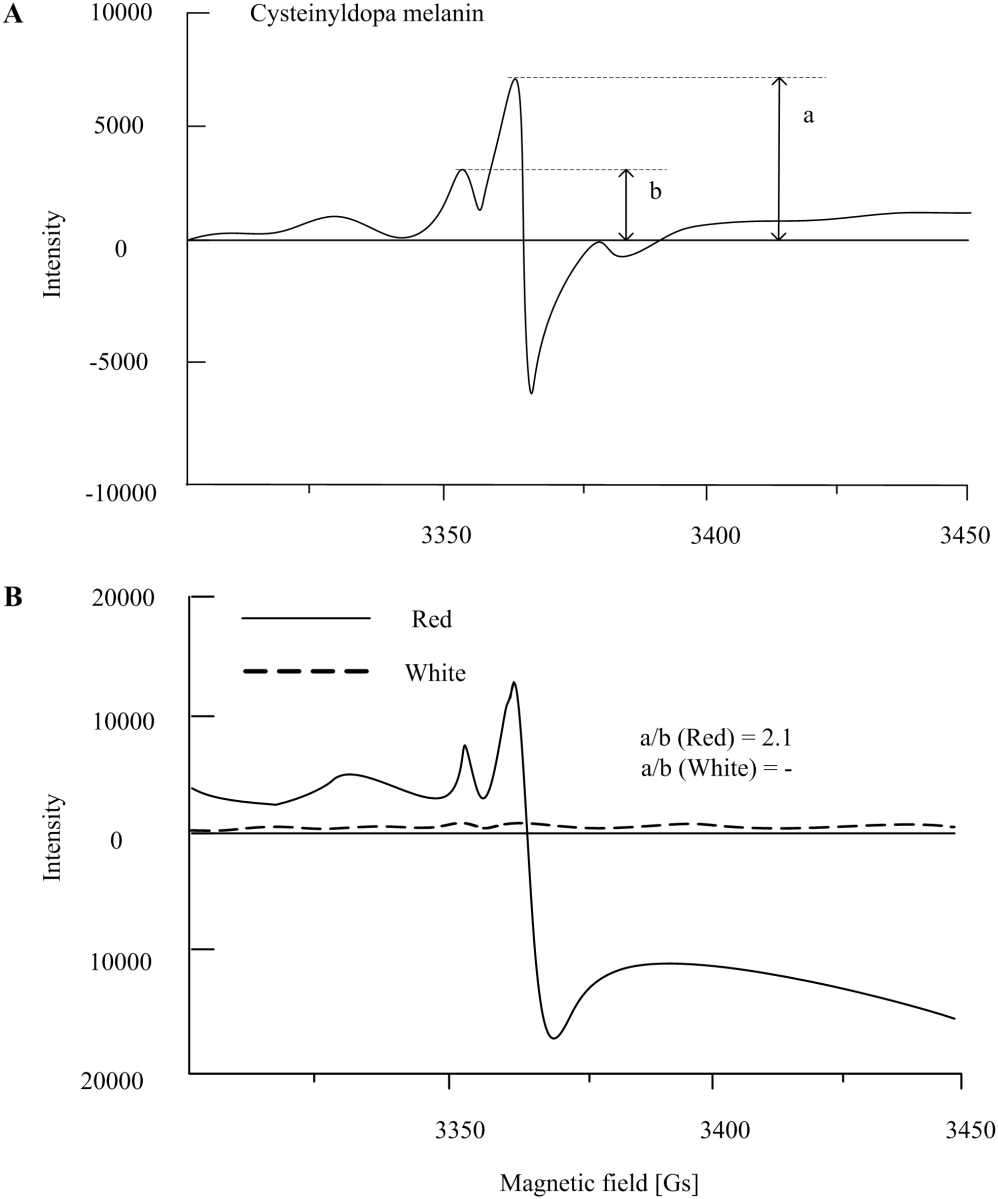
Quantitative real-time PCR (qPCR) analysis for melanogensis-associated genes expressed in the mantle of White and Red scallops.

The quantitative RT-PCR (qPCR) method was used to verify the RNA-Seq results, which resulted in the significantly higher expression of TYR-3 in the mantle tissue of White scallops than that of Red scallops (*p* < 0.01; Fig 3), in accordance with the RNA-Seq results. Furthermore, tissue expression pattern of TYR-3 was determined for both of Red and White scallops, showing the similar pattern of tissue-specific gene expression in the sampled tissues (data not show). The significantly highest expression was detected in the organ of shell formation—mantle tissue, whereas the lowest gene expression was observed in the tissues of foot and adductor muscle (*p* < 0.01).

In addition, other melanogensis-associated genes or pathways, including NOS (nitric oxide synthase), MITF (microphthalmia-associated transcription factor), TYR-1, CREB (cAMP responsive element binding protein), CBP (CREB binding protein), cGMP, sGC (soluble guanylate cyclase) and NMDA (Nmethyl-D-aspartate), were also selected to measure their relative mRNA expression in mantle tissues of Red and White scallops. Independent T-test indicated that there were six in eight genes (NOS, MITF, TYR-1, CREB, cGMP and sGC) detected significantly expressed between Red and White scallops (Fig 3). All of these genes were significantly higher expressed in the mantle of Red scallops than White scallops, except for TYR-1.

### Tyrosinase activities and tyrosinase protein level

**Fig 4:**
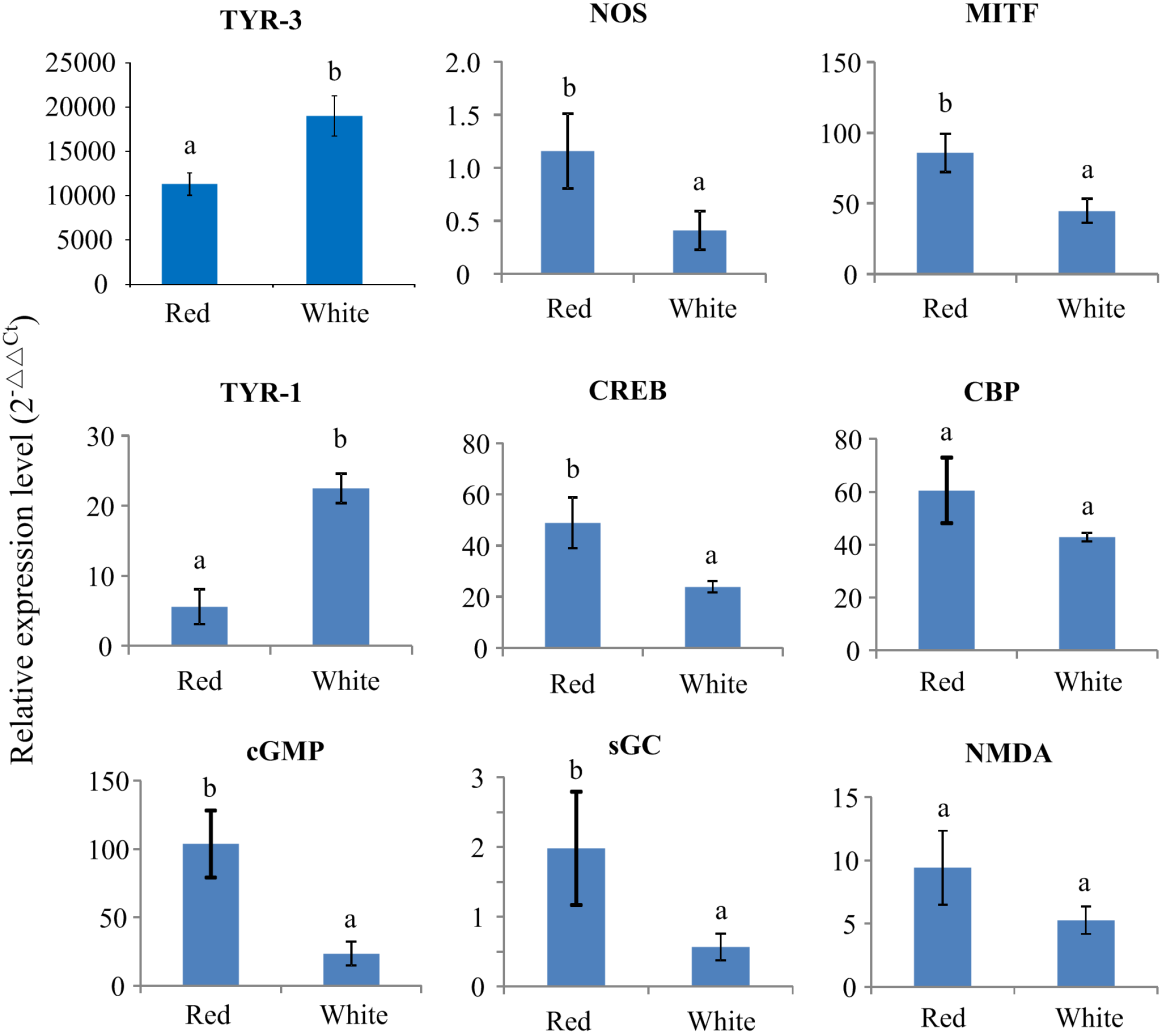
The quantification of tyrosinase activities and protein level (ELISA test) in the mantle of Red and White scallops.

The quantification of tyrosinase activities for the mantle of Red and White scallops was summarized in Fig 4. As a result, the value of tyrosinase activities (U/μg protein) in Red scallops (0.0853 ± 0.0065 U/μg protein) was approximately two-fold higher than that in White scallops (0.0427 ± 0.0020 U/μg protein; *P* < 0.01).

The concentration of tyrosinase protein in the mantle of Red and White scallops were determined by comparing the O.D. value of the samples to the standard curve made by the ELISA kit. As shown in Fig 4, the significantly higher protein concentration was detected in the Red scallops (36.6856 ± 1.4847 ng/L) than the White scallops (22.8507 ± 3.6868 ng/L; *P* < 0.05).

### Melanin determination by EPR measurement

**Fig 5:**
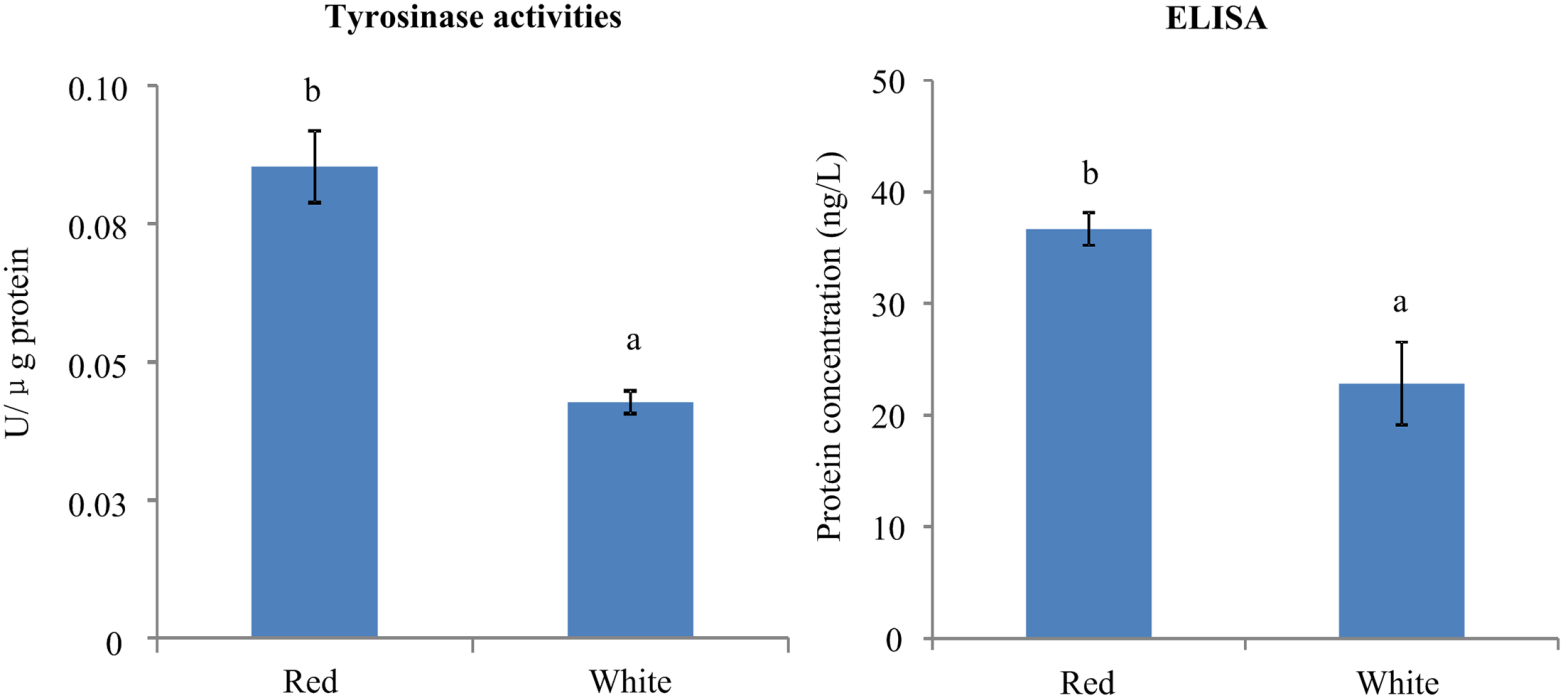
EPR spectra of cysteinyldopa melanin (10 mg/ml) and melanin extract from shell power of Yesso scallop (left shell; 7 g). All the samples were suspended in 50 mM zinc acetate and examined at 100K using the same apparatus parameters. a, height of major peak; b, height of the additional peak.

EPR measurement of the melanin standards and samples were summarized in Fig 5. The EPR signal of melanin was identified in the melanin extract of red shells, but it was too weak to be detected in the extract of white shells. As shown in Fig 5, the melanin samples from red shells have the similar EPR spectra with the synthetic pheomelanin (cysteinyldopa melanin). By the comparison of major and additional peak height, the ratio of eumelanin to pheomelanin (a/b) was calculated to be 2.1 in the melanin samples of red shells. However, the ratio of a/b could not be determined in the melanin samples of white shells due to their weak EPR signal.

## Discussion

In order to address one of the crucial issues of animal pigmentation that how gene expression correlates with melanin content, we have characterized the gene expression by DGE analysis in mantle tissue and determined the melanin content by EPR measurement in shells. The present results revealed a dozen of differentially expressed transcripts, which are potentially related to the color variation in this species. Compared to the Red scallops, the White scallops have the relatively lower mRNA expression of melanogensis-associated genes (except for TYR-1 and TYR-3), the weak EPR signal, lower concentration of tyrosinase protein, and decreased tyrosinase activity, which are suggested to be associated with the loss of melanin in the white shells. Our present findings shed new light on the role of transcriptional and post-transcriptional mechanisms in the regulation of tyrosinase activity and melanin synthesis in scallops.

For vertebrates, melanin is widely distributed in animal feathers, fur and skin, which is the main contributor to their pigmentation, especially in mammals and birds [7, 22, 37]. The two main types of melanin, red/yellow pheomelanin and brown/black eumelanin, are both derived from a common tyrosinase-dependent pathway [22]. Since tyrosinase is the rate-limiting enzyme of the melanin synthesis, the absence or dysfunction of tyrosinase and tyrosinase-related protein may result in the inability of melanocytes to make pigments, which causes preferential dilution of pheomelanin and shows albino phenotypes in mammals [9, 43]. The ratio of eumelanin and pheomelanin can be expressed quantitatively by EPR signal through the a/b [42, 44, 45]. In this study, the ratio of pheomelanin in total melanin (~30%) measured by EPR is similar with that in mammal skin and hair, which have a constant level of about 26% [23, 39, 44, 46]. EPR spectrum of the melanin extract from the Red scallops indicates that they contain a mixed type of pheomelanin and eumelanin in their pigmented shells. In contrast, melanin extract from the White scallops shows no detectable EPR signal, which indicates the occurrence of white shells due to lack of melanin.

Melanin synthesis is under complex regulatory control regulated by enzymes, structural proteins, transcriptional regulators, transporters, receptors, and growth factors in animals and microorganisms [37, 47]. Among them, tyrosinase is thought to be the key enzyme in the production of melanin. For bivalve molluscs, tyrosinase has been identified expressed in mantle tissue of the pearl oyster *Pinctada fucata* [48, 49], pacific oyster *Crassostrea gigas* [29], as well as the scallop *P*. *yessoensis* in our previous study [4]. Furthermore, localization of tyrosinase in pigmented regions has been reported in some bivalve species, such as pearl oyster *P*. *fucata* [28, 50], and hard clam *Mercenaria mercenaria* [51]. In this study, we have not only detected the EPR spectra of melanin from the shell extract of Red scallops, but also found moderately tyrosinase activity and protein level in their mantle tissue. These data provide new evidences for melanogenesis in scallops and consistent with the speculation that tyrosinase is probably secreted from the mantle tissue and transported to the calcified shell layer, where they contribute to melanin synthesis in bivalves [29, 50].

As reported, the process of melanin synthesis requires the control of multiple genes as well as their regulatory and structural products [37]. For tyrosinase, the posttranscriptional processing of pro-tyrosinase mRNA generates several alternatively spliced products, but only one transcript was able to confer tyrosinase enzyme activity [37, 52, 53]. In this study, we found the tyrosinase mRNA (TYR-3 and TYR-1) in both of Red and White scallops, but it showed the negative changes in relative abundances compared with tyrosinase protein and enzyme activity. The present data imply that the stimulation of tyrosinase activity may be caused by other alternatively spliced products, not TYR-3 and TYR-1 in the scallops. Therefore, it is speculated that the regulation of melanin synthesis though tyrosinase appears to be controlled by posttranslational mechanisms [37]. Additionally, for molluscs, hemocyanins and tyrosinase share the similar enzyme activation and catalysis, although their physiological functions differ [54]. In this study, it is therefore suggested that the increase of tyrosinase activity in Red scallops is caused by a hypothetical supplementation with the tyrosinase-like activity of hemocyanin, but not directly induced by the tyrosinase mRNA expression.

Besides tyrosinase, other important genes were also reported to be involved in the transcriptional regulation of the complex process of melanin synthesis, such as NOS, MITF, CREB, CBP, cGMP, sGC, NMDA and etc. [37]. For instance in skin pigmentation, ultraviolet B radiation acts through the stimulation of NOS and subsequent release of nitric oxide (NO), and then activation of sGC with subsequent accumulation of cGMP and protein kinase G to stimulate melanogenesis in the melanocytes [37, 55]. In addition, MITF plays a fundamental role in providing positive regulation of transcription of tyrosinase, TyrP1 and TyrP2 genes through interaction with M and E boxes, which may act as a self-regulating switchboard for diverse pathways regulating the activity of the melanogenic apparatus [56, 57]. The mechanism of cAMP regulation of melanogenesis involves the activation of protein kinase A, which involves phosphorylation of CREB and CBP [37]. NMDA receptors can interact with cGMP and MITF, resulting in activation of tyrosinase and increased melanin synthesis [26, 58]. In the present study, NOS, MITF, CREB, cGMP and sGC are significantly higher expressed in Red than White scallops, which are suggested to be responsible for the pigmentation in the red shells. These findings indicate that melanin synthesis in scallops may involve the transcriptional mechanism activated by different signaling systems [37].

Furthermore, the other differently expressed genes also provide some hints for shell color variation in the scallops (Table 2). For example, comp83659_c2 has the domain similarity with SRI (Set2 Rpb1 interacting), which mediates RNA polymerase II interaction and couples histone H3 K36 methylation with transcript elongation [59]. However, the provisional annotation sheds no light on the functional basis of the color variation or shell formation. In contrast, the unigene of comp86031_c0 is annotated related to conotoxins, which are small neurotoxic peptides with disulphide connectivity that modulate the activity of ion channels [60]. Also, the unigene of comp91852_c0 is suggested to be associated with the function of nucleic acid binding and zinc ion binding. These annotations suggest that these genes may be involved in biomineralization processes such as calcium channels and ion exchange during shell formation. All together, these findings are consistent with the current concept proposing that melanogenesis-controlling factors may be not arranged in simple sequences, but instead they usually interact in a complex and multidimensional network, which is determined by the genetic-biochemical-physical context [61].

**Table 2:** Identification of differentially expressed genes between the red and white of *Patinopecten yessoensis*.

Gene_id	T1 reads	C0 reads	log2FoldChange	*P*-adj	Discription
comp78910_c0	0.00	95.59	-	8.26E-12	-
comp82342_c0	5.41	161.43	-4.90	6.80E-10	-
comp86321_c0	11.53	198.74	-4.11	1.05E-08	-
comp91673_c0	198.70	28.04	2.83	8.39E-05	-
comp96387_c1	159.27	22.84	2.80	2.46E-04	-
comp54314_c0	148.00	21.37	2.79	2.97E-04	PREDICTED: hypothetical protein LOC100494154
comp51792_c0	5.71	79.09	-3.79	3.72E-04	-
comp94117_c0	9563.20	2405.38	1.99	2.19E-03	Putative tyrosinase-like protein tyr-3
comp83659_c2	15.36	150.75	-3.29	4.88E-03	SRI (Set2 Rpb1 interacting) domain
comp86031_c0	384.71	83.10	2.21	6.84E-03	Conotoxin//FHIPEP family
comp91852_c0	2531.43	639.72	1.98	9.59E-03	nucleic acid binding//zinc ion binding
comp77935_c0	0.44	55.19	-6.97	9.81E-03	-
comp78009_c0	67.36	8.00	3.07	9.81E-03	-
comp99770_c0	51.43	6.52	2.98	9.81E-03	-
comp98835_c0	198.60	42.93	2.21	1.05E-02	DNA polymerase (viral) C-terminal domain
comp81979_c0	1.83	44.42	-4.60	1.07E-02	-
comp96125_c0	97.58	7.33	3.73	1.18E-02	-
comp81217_c0	3.93	48.36	-3.62	1.24E-02	-
comp81333_c0	73.60	10.94	2.75	2.43E-02	-
comp93541_c0	4117.08	1267.84	1.70	2.49E-02	-
comp69580_c0	26.68	0.33	6.35	2.86E-02	-
comp53469_c0	170.76	20.77	3.04	3.10E-02	-
comp91333_c0	122.04	26.07	2.23	3.10E-02	Poly [ADP-ribose] polymerase 14
comp93725_c0	976.47	221.56	2.14	3.10E-02	-
comp87825_c0	88.71	20.06	2.14	3.71E-02	-

For bivalve molluscs, melanin pigmentation is almost certainly common in their shells, which probably forms in the secretory cells of the mantle edge and show as part of the general color mosaics in shells and is associated with photo-receptor mechanisms [15, 27, 28]. In cephalopod molluscs, they usually have a vivid, dynamic coloration due to the neurally controlled chromatophores in the body skin, which comprise elastic sacculus containing pigments to make visual signals for concealment and communication [62]. In *Octopus vulgaris*, there are some black chromatophores in their skin, which are suggested to be composed of melanin (eumelanin) [63]. Although the rapid changes of coloration of the cephalopod skin is neurogenic, the process of the melanin synthesis and its regulation, which undergoes a longer time regime, remains still dependent on the hormonal regulation [37, 62]. It is therefore suggested that the process of melanin synthesis in the shells of bivalves and the skin of cephalopods may have some similarities, since both of them are derived from tyrosinase [15, 63].

## Conclusion

In conclusion, the Illumina DGE analysis has revealed a dozen of differently expressed genes related to the color variation in *P*. *yessoensis*. EPR analysis indicates that EPR spectra of pheomelanin and eumelanin exist in the red shells, but no EPR signal of melanin was identified in white shells. With the integration of gene expression, EPR, ELISA and tyrosinase activity, our findings highlight the functional roles of melanogensis-associated genes in the melanization process of scallop shells, and shed new lights on the transcriptional and post-transcriptional mechanisms in the regulation of tyrosinase activity during the process of melanin synthesis. The present results will assist our molecular understanding of melanin synthesis underlying shell color polymorphism in scallops, as well as other bivalves, and also help the color-based breeding in shellfish aquaculture.
